# Physical Activity–Related Policy and Environmental Strategies to Prevent Obesity in Rural Communities: A Systematic Review of the Literature, 2002–2013

**DOI:** 10.5888/pcd13.150406

**Published:** 2016-01-07

**Authors:** M. Renée Umstattd Meyer, Cynthia K. Perry, Jasmin C. Sumrall, Megan S. Patterson, Shana M. Walsh, Stephanie C. Clendennen, Steven P. Hooker, Kelly R. Evenson, Karin V. Goins, Katie M. Heinrich, Nancy O’Hara Tompkins, Amy A. Eyler, Sydney Jones, Rachel Tabak, Cheryl Valko

**Affiliations:** Author Affiliations: Cynthia K. Perry, School of Nursing Oregon Health & Science University, Portland, Oregon; Jasmin C. Sumrall, Shana M. Walsh, Stephanie C. Clendennen, Robbins College of Health and Human Sciences, Baylor University, Waco, Texas, Megan S. Patterson, Baylor University, Waco, Texas; Steven P. Hooker, Arizona State University, Phoenix, Arizona; Kelly R. Evenson, Sydney Jones, University of North Carolina, Chapel Hill, Chapel Hill, North Carolina; Karin V. Goins, University of Massachusetts Medical School, Worcester, Massachusetts; Katie M. Heinrich, Kansas State University, Manhattan, Kansas; Nancy O’Hara Tompkins, West Virginia Prevention Research Center, West Virginia University, Charleston, West Virginia; Amy A. Eyler, Rachel Tabak, Cheryl Valko, Prevention Research Center, Brown School at Washington University in St. Louis, St. Louis, Missouri.

## Abstract

**Introduction:**

Health disparities exist between rural and urban residents; in particular, rural residents have higher rates of chronic diseases and obesity. Evidence supports the effectiveness of policy and environmental strategies to prevent obesity and promote health equity. In 2009, the Centers for Disease Control and Prevention recommended 24 policy and environmental strategies for use by local communities: the Common Community Measures for Obesity Prevention (COCOMO); 12 strategies focus on physical activity. This review was conducted to synthesize evidence on the implementation, relevance, and effectiveness of physical activity–related policy and environmental strategies for obesity prevention in rural communities.

**Methods:**

A literature search was conducted in PubMed, PsycINFO, Web of Science, CINHAL, and PAIS databases for articles published from 2002 through May 2013 that reported findings from physical activity–related policy or environmental interventions conducted in the United States or Canada. Each article was extracted independently by 2 researchers.

**Results:**

Of 2,002 articles, 30 articles representing 26 distinct studies met inclusion criteria. Schools were the most common setting (n = 18 studies). COCOMO strategies were applied in rural communities in 22 studies; the 2 most common COCOMO strategies were “enhance infrastructure supporting walking” (n = 11) and “increase opportunities for extracurricular physical activity” (n = 9). Most studies (n = 21) applied at least one of 8 non-COCOMO strategies; the most common was increasing physical activity opportunities at school outside of physical education (n = 8). Only 14 studies measured or reported physical activity outcomes (10 studies solely used self-report); 10 reported positive changes.

**Conclusion:**

Seven of the 12 COCOMO physical activity–related strategies were successfully implemented in 2 or more studies, suggesting that these 7 strategies are relevant in rural communities and the other 5 might be less applicable in rural communities. Further research using robust study designs and measurement is needed to better ascertain implementation success and effectiveness of COCOMO and non-COCOMO strategies in rural communities.

## Introduction

Rural residents have higher rates of chronic diseases and obesity than urban residents (1–5). Rural residents may have as much as 6.2% higher prevalence of obesity than urban residents (6,7). Physical inactivity is associated with higher rates of chronic diseases and obesity (7,8), and some research suggests that rural residents are less physically active than urban residents (9–11). Rural residents also have higher rates of poverty, fewer community resources, less access to preventive services and health care, greater geographic dispersion, and more transportation challenges (eg, lack of public transit, greater travel distance) than urban residents (12–18). Sixteen percent of Americans live in rural areas that encompass 72% of land in the United States. Although evidence supports the effectiveness of policy and environmental strategies to prevent obesity and promote health equity, much of this evidence is derived from nonrural settings (13,19,20).

In 2009, the Centers for Disease Control and Prevention (CDC) recommended 24 strategies for local communities to use in planning and monitoring obesity-related policy and environmental changes using preexisting data sources: the Common Community Measures for Obesity Prevention (COCOMO) (21). Twelve strategies focus on physical activity (PA) (Table 1): 4 strategies to “encourage physical activity or limit sedentary activity among children and youth” (strategy nos. 12–15) and 8 strategies to “create safe communities that support physical activity” (strategy nos. 16–23). The purpose of this study was to conduct a systematic literature review of the implementation, relevance, and effectiveness of physical activity–related policy and environmental strategies for obesity prevention in rural communities, including both COCOMO and non-COCOMO approaches. A secondary aim was to synthesize the evidence on the implementation success of the 12 physical activity–related COCOMO strategies in rural communities.

**Table 1:** Physical Activity–Related Strategies and Recommended Measurement Approaches From “Community Strategies and Measurements to Prevent Obesity in the United States”^a^

Strategy No.	Strategy and Recommended Measurement Approach
**Category: Encourage physical activity or limit sedentary activity among children and youth**	
**12**	**Require physical education in schools**
	Largest school district has a policy requiring number of PE minutes per week meeting physical activity recommendations
**13**	**Increase the amount of physical activity in physical education programs in schools**
	Largest school district has a policy that requires kindergarten–12 students to be active for at least 50% of time spent in PE classes
1**4**	**Increase opportunities for extracurricular physical activity**
	Percentage of schools in largest school district that allow use of athletic facilities by the public during nonschool hours
**15**	**Reduce screen time in public service venues**
	Licensed childcare facilities required to limit screen viewing time to ≤2 hours per day for children aged ≥2 years
**Category: Create safe communities that support physical activity**	
**16**	**Improve access to outdoor recreational facilities**
	Percentage of residential parcels located within ½ mile of ≥1 outdoor public recreation facility
**17**	**Enhance infrastructure supporting bicycling**
	Total miles of designated shared-use paths and bicycle lanes relative to total street miles (exclude limited access highways)
**18**	**Enhance infrastructure supporting walking**
	Total miles of paved sidewalks relative to total street miles
**19**	**Support locating schools within easy walking distance of residential areas**
	Largest school district has policy: new schools or fix existing schools in easy walking/bicycling distance of residential areas
**20**	**Improve access to public transportation**
	Percentage of residential and commercial parcels within ¼ mile of ≥1 bus stop or ½ mile of ≥1 train stop
**21**	**Zone for mixed-use development**
	Percentage of acres zoned for mixed use (residential with ≥1 public use)
**22**	**Enhance personal safety in areas where people are or could be physically active**
	Number of vacant or abandoned buildings relative to total number of buildings
**23**	**Enhance traffic safety in areas where people are or could be physically active**
	Local government policy for street design and operations with safe access for all users (include ≥1 complete streets element)

Abbreviation: PE, physical education.

^a^ Kettel Khan et al (21).

## Methods

### Data sources

A literature search was conducted in the following databases: PubMed, PsycINFO, Web of Science, Cumulative Index to Nursing and Allied Health Literature (CINHAL), and Public Affairs Information Service (PAIS). The search included articles published in English from 2002 through May 2013 and focused on findings from PA–related policy or environmental interventions. Each search used the following terms: “rural” AND “physical activity or exercise or sedentary or inactivity” AND “community or environment or policy.” Searches were repeated in a secondary literature search using search terms for Native American communities (“tribal” OR “reservation” OR “Native American” OR “indigenous”) and predominantly rural states. “Predominantly rural states” were identified where 1) most (half or greater) of the state was identified as rural using the Rural-to-Urban Continuum Codes and the Office of Management and Budget maps or 2) substantial portions of a state were identified as frontier using the Rural Assistance Center’s Frontier map (22,23). The following states were designated as predominantly rural: Alaska, Idaho, Kansas, Maine, Mississippi, Montana, Nebraska, Nevada, New Hampshire, New Mexico, North Dakota, Oklahoma, Oregon, South Dakota, Texas, Utah, Vermont, West Virginia, and Wyoming. Relevant references cited in each identified study were also included in the secondary literature search. Methods mirrored a companion literature review describing application of nutrition-related COCOMO strategies in rural communities (24).

### Study selection

At least 2 researchers reviewed titles, abstracts, and complete texts of articles for inclusion. Studies were included that reported findings from empirical formative, process, or outcome research with strategies aimed at changing policy or environments to support PA in rural US or Canada communities. Publications were excluded if 1) both rural and urban communities were included and rural-specific findings were not reported, 2) the primary focus was on instrument development or individual-level behavioral change, or 3) studies were descriptive or did not describe an intervention.

### Data extraction

The article extraction team consisted of 18 trained researchers. Data for each article were extracted independently by 2 trained researchers. We used a customized Qualtrics (Qualtrics LLC) online survey as a tool to extract information about study setting, geographic location, sample characteristics, rural definition, design, methods, results, and bias-risk assessment (25–27). After independent extraction, results were compared and discrepancies were resolved by consensus. Study quality was examined for randomized control trials (RCTs) using Cochrane Collaboration’s assessment tool. We used GRADE guidelines of bias risk for observational studies to assess non-RCTs, including formative studies, because the Cochrane tool focuses only on RCTs (25–27). The Cochrane tool assesses risk of bias across 6 categories: sequence generation, allocation concealment, blinding, incomplete outcome data, selective outcome reporting, and other sources of bias (25,26); all categories were assessed as designed. GRADE guidelines assess risk of bias across 4 categories: appropriate eligibility criteria, measurement of exposure and outcome, control of confounding, and incomplete follow-up (27); all categories were assessed as designed. Risk of bias was rated as low (score of 1), high (score of 0), or unclear (score of 0) for each Cochrane or GRADE category based on study type (25); overall summary scores for bias risk were calculated and categorized as low, medium, or high (RCTs: low risk = 5 or 6, medium risk = 2–4, and high risk = 0 or 1; non-RCTs: low risk = 3 or 4, medium risk = 2, and high risk = 0 or 1). Extraction data entered into Qualtrics were downloaded into Excel for synthesis. We organized data into the following categories: 1) study location, setting, approach, and bias-risk assessment; 2) COCOMO strategies used; 3) non-COCOMO strategies used; 4) measurement of policy and environmental strategies; and 5) intervention effects on policy, environment, behavioral, and health outcomes.

## Results

Searches returned 9,879 articles, of which 2,002 were identified as relevant for further screening based on title and abstract. Duplicates were removed, leaving 488 records for full-text screening; 443 of these did not meet inclusion criteria. The remaining 45 articles represented 41 distinct studies and were assigned for data extraction; 15 articles were excluded during extraction for various reasons (Figure). Thus, 30 articles representing 26 distinct studies were included in the final synthesis.

**Figure:**
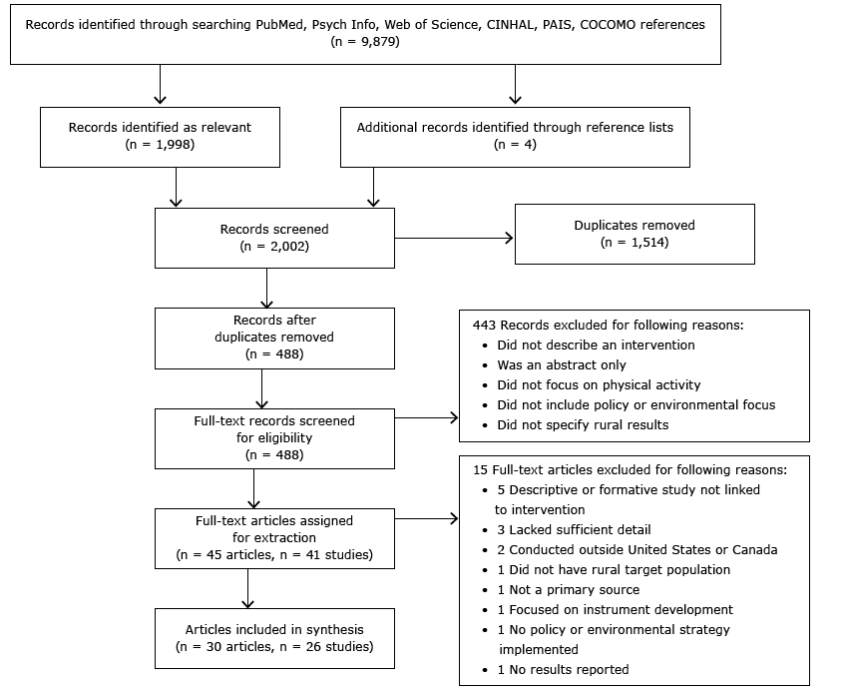
Preferred Reporting Items for Systematic Reviews and Meta-Analysis (PRISMA) flow diagram for study inclusion in a systematic review of physical activity–related policy and environmental strategies for obesity prevention in rural communities. Abbreviations: CINAHL, Cumulative Index to Nursing and Allied Health Literature; PAIS, Public Affairs Information Service; COCOMO, Common Community Measures for Obesity Prevention (21).

### Study location, setting, approach, and bias-risk assessment

Of the 26 studies, 3 were conducted in Canada and 23 in the United States; 4 studies were conducted with American Indian tribes or First Nations of Canada (Table 2). Rural location of 19 studies was defined by authors as “rural,” “small town,” or “remote”; 4 studies provided information about population density to define rurality, and 3 were identified as rural only through descriptions of tribal or reservation areas. Study settings included schools (n = 18), communities (defined broadly without identification of an entity, organization, or institution; n = 12), worksites (n = 5), churches (n = 1), homes (n = 2), and childcare (n = 1); 5 interventions targeted multiple settings. In the 18 school-setting studies, interventions resulted in changes that affected students (n = 14), changes in the use of facilities for the community (n = 3), or changes that affected employees (n = 1). Study designs included formative (n = 7), process (n = 16), or outcome (n = 20) evaluations; 15 included 2 or more types of evaluation. Only 3 studies were RCTs. Bias-risk assessments showed that 19 studies had high risk of bias, 4 studies had medium risk, and 3 studies had low risk (Table 3). None of the 23 non-RCTs adequately controlled for confounding, and 5 non-RCTs had flawed measurement. Six non-RCTs developed and applied appropriate eligibility criteria, and 7 non-RCTs had complete follow-up. Of the 3 RCTs, one had medium risk and 2 had high risk of bias (Table 3). Sequence generation was absent in all RCTs, and all reported selective outcome data and had other sources of bias.

**Table 2:** Location, Setting, COCOMO Strategies and Non-COCOMO Strategies Used, and Study Evaluation Focus in Review of Studies on Physical Activity–Related Policy and Environmental Strategies to Prevent Obesity in Rural Communities, 2002–2013

Study	Location	Setting	COCOMO Strategy	Non-COCOMO Strategy	Evaluation Focus
Bachar et al (28)	Western North Carolina, EBCI American Indian Reservation	School (students), community, worksite, church	No. 14	Adopt worksite policies or practices.	Outcome
Belansky et al (29)	Colorado	School (students)	Nos. 12, 13	Increase PA opportunities at school outside of PE.	Outcome
Belansky et al (30)	South central Colorado	School (students)	Nos. 13, 18	Increase PA opportunities at school outside of PE; increase amount of and access to PA equipment or improve existing equipment resources.	Process, outcome
Caballero et al (31), Davis et al (32), Going et al (33), Steckler et al (34)	Schools serving American Indian communities in Arizona, New Mexico, South Dakota	School (students)	Nos. 12, 13	Increase PA opportunities at school outside of PE.	Process, outcome
Devine et al (35)	Upstate New York	Worksite	None	Promote PA resources.	Process
Drummond et al (36)	Yuma County, Arizona	Childcare setting	No. 15	Increase amount of and access to PA equipment or improve existing equipment resources; reduce sedentary time in school or preschool setting.	Process, outcome
DyckFehderau et al (37)	Alberta, Canada, main reserve land of the Alexander First Nation	Community	Nos. 16–18	Increase amount of and access to PA equipment or improve existing equipment resources.	Formative
Farag et al (38)	Southwestern Oklahoma	School (employees)	No. 18	Increase amount of and access to PA equipment or improve existing equipment resources; promote PA resources; adopt worksite policies or practices.	Process, outcome
Filbert et al (39)	Jefferson County, Kansas	School (students)	Nos. 12, 14, 18	None	Formative, outcome
Friesen (40)	Wells County, Indiana	School (facility), community	No. 18	Provide access to public buildings after hours; promote PA resources.	Outcome
Gantner and Olson (41)	Upstate New York	Community	None	Promote PA resources.	Process
Gombosi et al (42)	Tioga County, Pennsylvania	School (students), community, worksite, home	No. 14	Adopt PA-supportive curriculum in school district.	Outcome
Humbert and Chad (43)	Saskatchewan Province, Canada	School (students)	Nos. 12–14	None	Process
Laing et al (44)	Mason County, Washington	Worksite	None	Adopt worksite policies or practices.	Process, outcome
Martin et al (45)	Maine	School (students), school (facility), community, worksite	Nos. 14, 17, 18	Increase amount of and access to PA equipment or improve existing equipment resources.	Process, outcome
Ndirangu et al (46)	Lower Mississippi Delta Region, Arkansas, Louisiana, Mississippi	Community	Nos. 16, 18, 22, 23	Provide access to public buildings after hours; promote PA resources.	Formative
Pate et al (47)	South Carolina	School (students)	No. 14	Increase PA opportunities at school outside of PE.	Process, outcome
Jilcott Pitts et al (48)	Lenoir County, North Carolina	Community	Nos. 12–23	None	Formative
Reger-Nash et al (49)	North central West Virginia	Community	No. 18	Promote PA resources.	Process, outcome
Riley-Jacome et al (50)	Columbia and Greene counties, New York	School (facility)	None	Provide access to public buildings after hours.	Formative, process, outcome
Schetzina et al (51)	Northeastern Appalachian Tennessee	School (students)	Nos. 16, 18	Increase PA opportunities at school outside of PE.	Formative, process, outcome
Schetzina et al (52)	Northeastern Appalachian Tennessee	School (students)	Nos. 16, 18	Increase PA opportunities at school outside of PE.	Process, outcome
Story et al (53)	Pine Ridge Reservation, South Dakota	School (students), home	Nos. 13–15	Increase PA opportunities at school outside of PE; increase amount of and access to PA equipment or improve existing equipment resources; reduce screen time at home.	Formative, outcome
Tomlin et al (54), Naylor et al (55)	Northwestern British Columbia, Canada	School (students)	Nos. 12, 14	Increase PA opportunities at school outside of PE.	Process, outcome
Wiggs et al (56)	Southeast Missouri (Ozarks)	Community	Nos. 16, 18	None	Process, outcome
Williamson et al (57)	Louisiana	School (students)	No. 13	None	Outcome

Abbreviations: COCOMO, Common Community Measures for Obesity Prevention (21); EBCI, Eastern Bank of Cherokee Indians; PA, physical activity; PE, physical education.

**Table 3:** Description of Research Design and Results of Process or Outcome Evaluations After Implementation of Physical Activity Interventions, Review of Studies on Physical Activity–Related Policy and Environmental Strategies to Prevent Obesity in Rural Communities, 2002–2013

Study/Design and Bias Risk^a^	Reach,^b^ Sample Size^c^ and Setting	Factors Influencing Intervention Implementation^d^	Policy or Environmental Change Implemented	Changes Effected in Target Population	
**Bachar et al, *Preventing Chronic Disease*, 2006 (** 28 **)**					
Pre–post; no comparison group; high bias risk (score, 0)	**Reach**: 1 tribal elementary school (up to 600 students enrolled); 1 tribal workplace; 5 churches. **Sample**: N = 86 employees; N = 140 students.	—	Employees given time off to exercise; increase in opportunities PA for students; student awardees given swim party instead of pizza party.	**Psychosocial**	School participants increased awareness of necessity to be physically active (teacher interview).
				**Behavior**	Increase in PA of worksite participants (self-reported in client histories and interviews; tool not specified).
				**Health status**	71% of worksite participants lost weight (objectively measured) and decreased their BMI (objectively measured); some participants self-reported (in interviews) improvements in chronic illness (eg, decreased or eliminated diabetes medications, high blood pressure medications, or both).
**Belansky et al, *Journal of Public Health Policy*, 2009 (** 29 **)**					
Pre–post follow-up; no comparison group; high bias risk (score, 1)	**Reach: **40 school districts. **Sample**: N = 45 elementary schools (mean number of enrolled students = 204).	**Barriers:** competing pressures, lack of resources devoted to local wellness policy, principals’ unfamiliarity with local wellness policy, lack of a champion, lack of accountability mechanisms. **Facilitators of success:** committee of diverse individuals inside and outside of the school to develop policies.	Increase in mean minutes of PE (PE teacher’s self-report in survey); decrease in time for recess (principal’s self-report in survey); no increase in number of principals requiring teachers to allow students to participate in PE or recess when incivilities occur (principal’s self-report in survey); most principals were not familiar with local wellness policy; most local wellness policies had weak wording.	**Psychosocial**	—
				**Behavior**	—
				**Health status**	—
**Belansky et al, *Journal of School Health*, 2013 (** 30 **)**					
Pair randomized; medium bias risk (score, 2)	**Reach: **13 elementary schools. **Sample**: 10 elementary schools (mean number of enrolled students = 203).	**Barriers:** lack of teacher and staff buy-in, principal turnover. **Facilitators of success:** involve staff at all levels, funding, communication within organization, timely feedback.	2 schools increased PE class time (eg, smaller classes, comprehensive curriculum); 4 schools made changes to recess (eg, organized activities during recess); 4 schools made changes to the playground (eg, balls, markings for 4-square, walking track).	**Psychosocial**	—
				**Behavior**	—
				**Health status**	—
**Caballero et al, *American Journal of Clinical Nutrition*, 2003 (** 31 **); Davis et al, *Preventive Medicine*, 2003 (** 32 **); Going et al, *Preventive Medicine*, 2003 (** 33 **); Steckler et al, *Preventive Medicine*, 2003 (** 34 **)**					
RCT (school level); high bias risk (score, 1)	**Reach:** 41 schools in 7 American Indian communities (2,059 students). **Sample:** baseline N = 1,704 children; post N = 1,409 children.	—	By year 3, all schools offered PE 3 times per week, and 56% of schools offered PE 5 days per week. Average of 1.6 activity breaks per school day.	**Psychosocial**	Increase in PA self-efficacy (self-report in survey).
				**Behavior**	No significant differences in PA change between groups for subset using 1 day of accelerometer data (n = 278), although nonsignificant increases in PA were found for intervention group (accelerometer, TriTrac-R3D); significantly higher self-reported PA at post-test for intervention schools (self-report: 24-hr PA recall survey).
				**Health status**	No significant differences between intervention and control groups for all anthropometric variables (objectively measured: BMI; % body fat using bioelectrical impedance; skinfold thickness).
**Devine et al, *Evaluation and Program Planning*, 2012 (** 35 **)**					
Pre–post; mixed methods process evaluation; no comparison group; high bias risk (score, 0)	**Sample**: N = 226 women at 5 worksites	**Facilitators to adherence**: accountability to coworkers and public displays of walking achievements. Reach at each worksite ranged from 19%–96%.	Worksite walking program, maps of walking trails at worksite.	**Psychosocial**	Increase in awareness of walking (self-reported in focus groups).
				**Behavior**	—
				**Health status**	—
**Drummond et al, *Health Promotion Practice*, 2009 (** 36 **)**					
Pre–post; no comparison group; high bias risk (score, 1)	**Reach**: 30 child care settings, serving 1,876 children. **Sample:** N = 17 of the 30 child care centers.	**Facilitators:** program was accredited as continuing education for childcare providers, accreditation provided incentive for home-based childcare centers.	Increase in number of centers that had portable play equipment, had indoor play space for running, did not restrict PA as a punishment, and implemented PA best practices; increase in percentage of centers providing all children with daily PA time (active play time ≥60 min, ≥2 outdoor active play times, based on staff self-report).	**Psychosocial**	—
				**Behavior**	Increase in level of staff member PA (informal self-report from site coordinator).
				**Health status**	—
**Farag et al, *BMC Public Health*, 2010 (** 38 **)**					
Pre–post; no comparison group; high bias risk (score, 0)	**Reach**: 1 school district with 5 schools. **Sample:** baseline N = 202 employees; post N = 187.	—	Worksite wellness program implemented. Employees could use planning period to exercise; treadmills added in schools; hallways marked with mileage.	**Psychosocial**	—
				**Behavior**	Nonsignificant increase in PA: increase in mean MET minutes per week (self-reported in survey: IPAQ-short).
				**Health status**	Significant decrease in total, HDL and LDL cholesterol levels (objectively measured) and decrease in systolic blood pressure (objectively measured).
**Filbert et al, *Journal of Community Health Nursing*, 2009 (** 39 **)**					
2-Phase study. Phase 1: retrospective observation. Phase 2: implementation school health program. High bias risk (score, 0)	**Reach**: 5 school districts. **Sample**: Phase 1: N = 174 (78 girls and 96 boys).	—	Built walking trail for student and community use, maintained daily PE, implemented employee wellness program.	**Psychosocial**	—
				**Behavior**	—
				**Health status**	—
**Friesen, *Family and Consumer Sciences Research Journal*, 2010 (** 40 **)**					
Annual cross-sectional assessment for 4 years; pre–post; no comparison group; high bias risk (score, 0)	**Reach**: 1 community, 3 school districts (10 schools), 52 worksites. **Sample:** N = 1,666 (annual community survey); adult PA program participants (N = 226); worksite wellness participants (N = 333).	—	School wellness policies developed and implemented; school facilities opened to community in all 10 schools; centralized walking path built on county fairgrounds.	**Psychosocial**	Adult PA program participants showed improvement in readiness to engage in PA for 30 min 5 days per week (self-reported in survey).
				**Behavior**	Community survey participants showed significant increase in days per week of PA (self-reported in survey, tool not specified); adult PA program participants showed increase in percentage engaged in 30 min per day of PA 3 to 7 days per week after 1 semester (self-reported in survey, tool not specified).
				**Health status**	139 Worksite wellness participants lost on average 3 pounds after 1 semester and 63 participants lost on average 5 pounds after 2 semesters (objectively measured).
**Gantner and Olson, *Evaluation and Program Planning*, 2012 (** 41 **)**					
Cross-sectional (initial year and year 2); qualitative; high bias risk (score, 0)	**Reach**: 8 counties. **Sample:** N = 20 community partners; N = 31 survey participants at baseline, N = 20 survey participants at year 2; N = 21 interviews.	Identified barriers: lack of organizational support for policy change, lack of political power to make change, need to develop skills and knowledge, frustration with long-term timeframe to make change, lack of consistent funding over long-term	Creation of distribution of map of PA opportunities in county.	**Psychosocial**	Increase in awareness of how to advocate for policy and environment change (self-report by partnership members in interviews).
				**Behavior**	—
				**Health status**	—
**Gombosi et al, *Clinical Pediatrics*, 2007 (** 42 **)**					
Nonrandomized age-matched cohorts; high bias risk (score, 0)	**Reach**: 6,000 students in preschool through high school with an annual cohort of approximately 4,800 K–8 school children; 7 worksites, 15,000 employees; and 1,000 residents of 8 communities.	—	Worksite wellness program, health curriculum implemented in schools; community PA events implemented.	**Psychosocial**	—
				**Behavior**	—
				**Health status**	Increase in prevalence of overweight and obesity (measurement method not specified).
**Humbert and Chad, *Avante*, 2003 (** 43 **)**					
Two-year longitudinal; qualitative; high bias risk (score, 1)	**Reach:** 5 schools: one K–3 school (75 students) and 5 K–6 schools (range, 100–300 students per school). **Sample**: 50 teachers and 5 administrators.	**Facilitators and recommendations:** Quality PE most critical component; need widespread support from school and community, adequate time for implementation, teacher effort and team work.	Schools offered daily PE; increase in opportunities for PA during school.	**Psychosocial**	Increase in awareness of importance of PA among administration and teachers.
				**Behavior**	—
				**Health status**	—
**Laing et al, *Preventing Chronic Disease*, 2012 (** 44 **)**					
Pre–post; no comparison group; medium bias risk (score, 2)	**Reach: **23 worksites (average 42 workers per worksite). **Sample: **23 employers.	Factors influencing worksite participation: upper management support and concern for health needs of employees. Easy to implement and broad in scope.	Significant increase in best practices implemented; increase in number of employers offering PA programming; increase in implementation of PA policies.	**Psychosocial**	Increase in awareness of opportunities for PA.
				**Behavior**	—
				**Health status**	—
**Martin et al, *Preventing Chronic Disease*, 2009 (** 45 **)**					
Retrospective evaluation; high bias risk (score, 0)	**Sample**: N = 31 Community agency–school partnerships	—	1,683 Environmental changes supporting PA were accomplished, including new walking or biking trails, employee wellness committees, community access to PA equipment.	**Psychosocial**	—
				**Behavior**	—
				**Health status**	—
**Pate et al, *American Journal of Health Promotion*, 2003 (** 47 **)**					
Nonrandomized; 2-groups’; pre–post; low bias risk (score, 3)	**Sample:** N = 434 students in grade 5	Community ownership of program not achieved; only after-school summer programming implemented as planned; transportation had impact on attendance; only 5% of children attended at least 50% of sessions; staff training took longer than expected; staff did not understand concept of self-efficacy or emphasis on noncompetitive programming	Increase in opportunities for PA.	**Psychosocial**	No change in beliefs about PA intention, PA consequences, social influences on PA, or PA self-efficacy (self-reported in surveys).
				**Behavior**	No change in moderate-to-vigorous PA levels (self-reported using a previous day PA recall survey for after-school PA time).
				**Health status**	—
**Reger-Nash et al, *Journal of Physical Activity & Health*, 2008 (** 49 **)**					
Nonrandomized; 2-group; pre–post; low bias risk (score, 3)	**Reach**: 5,400 residents. **Sample**: baseline survey: N = 1,233 adults in intervention community and N= 633 in comparison community. Follow-up survey: N = 887 in intervention and N = 446 in comparison	Successful media campaign: 1,143 television reports, 167 radio reports, 104 print media reports, and 17 campaign-related photos in newspapers.	Increase in funding for trail maintenance and sidewalk construction: increase in opportunities for PA in community (walking league).	**Psychosocial**	—
				**Behavior**	Significant increase in community members being sufficiently active (self-reported in survey: BRFSS).
				**Health status**	—
**Riley-Jacome et al, *Journal of Primary Prevention*, 2010 (** 50 **)**					
Post only; no comparison group; high bias risk (score, 0)	**Reach**: 3 school districts. **Sample**: N = 40 survey responses; N = 55 completed walking logs.	Existing school insurance policies were sufficient for community walking program; no additional school staff time required. Barriers: distance to school buildings, conflicts with school related activities, lack of person to administer program.	Increase in opportunity for PA; opened up schools for community walking program.	**Psychosocial**	Increase in social support (self-reported in focus groups).
				**Behavior**	Increase in level of PA (self-reported in survey: 1 item asking participants to recall change in PA).
				**Health status**	—
**Schetzina et al, *Family & Community Health*, 2009 (** 51 **)**					
Pre–post; no comparison group; high bias risk (score, 0)	**Reach: **1 elementary school. **Sample**: N = 114 students in grade 3 or grade 4 and 40 teachers.	Some teachers reported pedometers cumbersome to use; 87% of teachers reported program acceptability.	Indoor and outdoor walking trails established; instituted “move it moments” (5 min of PA); all teachers reported using “move it moments”; most teachers reported most or all students wore pedometers.	**Psychosocial**	Teachers perceived “move it moments” improved student behavior during class.
				**Behavior**	Significant increase in steps per day (pedometer).
				**Health status**	No change in BMI *z* scores in first 7 months of the program; students with healthy weight or at risk for overweight (85th–<95th percentile) were more likely to decrease BMI *z* score; students who were overweight were more likely to show no change in BMI z score than were healthy-weight or at-risk-for-overweight students.
**Schetzina et al, *Family & Community Health*, 2011 (** 52 **)**					
4-Year follow-up; pre–post; high bias risk (score, 0)	**Reach: **1 elementary school. **Sample:** N = 66 students in grade 4 and 23 teachers.	—	Indoor and outdoor walking trails established; 86% of teachers reported using pedometers in class; 91% of teachers reported using “move it moments” (5 min of PA) daily in last month; 14% of teachers reported using indoor walking trails weekly as part of program (self-reported in survey).	**Psychosocial**	No significant change in descriptive family norms or descriptive or injunctive friend norms (self-reported in survey)
				**Behavior**	Significant increase in steps at the school-level (pedometers).
				**Health status**	—
**Story et al, *Obesity*, 2012 (** 53 **)**					
RCT; pre–post (school level); high bias risk (score, 0)	**Reach:** 14 schools. **Sample:** N = 232 boys and 222 girls in kindergarten or 1st grade.	—	Classroom action breaks; outside class walks; modified PE class; family activities.	**Psychosocial**	—
				**Behavior**	No significant changes in PA in schools (teacher self-report of school time spent in PA).
				**Health status**	10% Decrease in prevalence of overweight (based on objective measurements of height and weight).
**Tomlin et al, *International Journal of Circumpolar Health*, 2012 (** 54 **); Naylor et al, *Rural and Remote Health*, 2010 (** 55 **)**					
Pre-post; no comparison group; low bias risk (score, 3)	**Reach:** 3 schools. **Sample:** N = 148 students.	Barriers: lack of time and school resources, high staff turnover, evaluation requirements, student behavior, low levels of staff knowledge about healthy living. Facilitators: training, resources, and ease of implementation.	Action bins distributed to classrooms for use with 15 min action break each day; increase in PA opportunities.	**Psychosocial**	—
				**Behavior**	No significant change in moderate-to-vigorous PA for self-report or subset (n = 30) using ≥3 days of accelerometer data (self-reported in survey: PAQ; subset accelerometers: Actigraph GT1M).
				**Health status**	No change in BMI (based on objective measurements of height and weight); increase in aerobic fitness (20-meter shuttle run).
**Wiggs et al, *Health Promotion Practice*, 2008 (** 56 **)**					
Case study; retrospective evaluation (post only); qualitative; high bias risk (score, 0)	**Reach:** Several counties.	Issues to consider in trail development: plan for maintenance trail, funding sources, location, size, objectives, recognition of funding sources on trail. Liability concerns were not an issue.	Construction of 30 walking trails in multiple counties, with most in residential park areas.	**Psychosocial**	—
				**Behavior**	Increase in the walking time of most walkers since using trail (interviews), increase in percentage of trail users’ PA since using the trail (self-reported in random-digit–dialed telephone survey: 1 item asking participants to recall change in PA since using trails) (58)
				**Health status**	—
**Williamson et al, *Obesity*, 2012 (** 57 **)**					
Longitudinal (pre–post, month 18, month 28); cluster RCT (school systems were clustered); 2 intervention groups and 1 control group; medium bias risk (score, 2)	**Reach**: 4, 857 students in grades 4–6. **Sample:** students in grades 4–6; N = 713 in primary intervention, N = 760 in primary and secondary intervention; and N = 587 in control.	—	Modified PE program	**Psychosocial**	—
				**Behavior**	No changes in PA or sedentary behavior (self-reported in survey: SAPAC).
				**Health status**	No changes in percentage body fat or BMI between intervention groups (based on objective measurements of height and weight); decrease in percentage body fat among boys, a slower increase in percentage body fat among girls in environmental-change group than in control, and significantly smaller increases in BMI for white girls between environmental-change group and control group at month 28 (based on objective measurements of height, weight, and body fat).

Abbreviations: —, data not reported; BMI, body mass index; BRFSS, Behavioral Risk Factor Surveillance System; HDL, high-density lipoprotein, IPAQ–Short, International Physical Activity Questionnaire–Short Form; LDL, low-density lipoprotein; PA, physical activity; PE, physical education; RCT, randomized control trial; MET, metabolic equivalent, PAQ, Physical Activity Questionnaire for Children and Adolescents; SAPAC, Self-Administered Physical Activity Checklist.

^a^ Bias risk was determined using Cochrane Collaboration’s assessment tool for RCTs and GRADE guidelines for non-RCTs (25–27). The Cochrane tool assesses risk of bias across 6 categories: sequence generation, allocation concealment, blinding, incomplete outcome data, selective outcome reporting, and other sources of bias (25,26); GRADE guidelines assess risk of bias across 4 categories: appropriate eligibility criteria, measurement of exposure and outcome, control of confounding, and incomplete follow-up (27). Risk of bias was rated as low (score of 1), high (score of 0), or unclear (score of 0) for each Cochrane or GRADE category based on study type (25); overall summary scores for bias risk were calculated and categorized as low, medium, or high (RCTs, low risk = 5 or 6, medium risk = 2–4, and high risk = 0 or 1; non-RCTs: low risk = 3 or 4, medium risk = 2, and high risk = 0 or 1).

^b^ When reported, we listed reach, which is the number of community members potentially affected by an intervention.

^c ^When reported, we listed the sample size of participants who completed evaluation measures for each study.

^d^ When reported, we listed the factors influencing intervention implementation.

### Use of COCOMO strategies

Although only one study referenced CDC’s COCOMO strategies (48), 22 of the 26 studies applied at least one PA-related COCOMO strategy (Table 2), and 4 studies did not apply any COCOMO strategies. The mean number of COCOMO strategies applied was 2.0 (standard deviation [SD], 2.3; range, 0–12). The 2 most commonly applied COCOMO strategies were no. 18, “enhance infrastructure supporting walking” (n = 11), and no. 14, “increase opportunities for extracurricular physical activity” (n = 9). Fourteen studies applied at least one COCOMO strategy to “encourage physical activity or limit sedentary activity among children and youth,” 12 studies applied at least one COCOMO strategy to “create safe communities that support physical activity,” and 4 studies applied at least one of each. Identified barriers to implementing these strategies in rural areas were staff turnover and lack of staff buy-in, organizational or community support, resources, and political will. Identified facilitators were communication, accountability, training, and ease of implementation.

### Use of non-COCOMO strategies

One or more non-COCOMO strategies were mentioned in 21 studies (Table 2). Four studies incorporated only non-COCOMO strategies and 6 studies incorporated 2 or more non-COCOMO strategies. The mean number of non-COCOMO strategies applied was 1.1 (SD, 0.8). Eight non-COCOMO strategies were identified: increase PA opportunities at school outside of physical education (PE) (n = 8) (eg, classroom activity breaks, longer school recess, reversing lunch and recess); increase amount of and access to PA equipment or improve existing equipment resources (n = 6); promote PA resources (n = 6) (eg, signs to promote walking routes or trails); provide access to public buildings after hours for PA purposes (n = 3); adopt worksite policies or practices (n = 3) (eg, allowing PA breaks during workday); reduce home screen time (n = 1); reduce school or preschool sedentary time (n = 1); and school district-wide adoption of a PA-supportive curriculum (n = 1). The mean number of COCOMO and non-COCOMO strategies applied per study was 3.1 (SD, 2.3).

### Measurement of policy and environmental strategies

Measurement of policy or environmental changes was not consistent or standard across studies, and researchers often did not use COCOMO-suggested measurements (Table 3). For example, studies (n = 6) that “increased the amount of physical activity in PE programs in schools” (strategy no. 13) documented results by indicating use of a modified PE program, increased minutes in PE or increased time in PA during PE (29–34,43,53,57). Some studies used a similar non-COCOMO metric to measure change. For example, the 8 studies that “increased opportunities of extracurricular physical activity” (strategy no. 14) measured change by indicating the presence of increased opportunities for PA (28,39,42,43,45,47,53–55).

### Intervention effects on policy, environment, behavioral, and health outcomes

Sixteen interventions had at least one positive environmental change or result, and 18 interventions reported a positive policy change or result (Table 3). Three studies focused solely on formative approaches without reporting policy or environmental results (Table 4). Seven of the 8 nonformative studies that “required PE” (strategy no. 12) or “increased amount of physical activity in PE programs” (strategy no. 13) reported a positive PE policy or environmental change (Table 3). All 3 studies that adopted worksite policies promoting PA documented policy implementation, and 2 studies measured improvements in health status (28,38).

**Table 4:** Description of Results of Formative Evaluations, Review of Studies on Physical Activity–Related Policy and Environmental Strategies to Prevent Obesity in Rural Communities, 2002–2013

Study	Design	Sample Size and Setting (If Reported)	Policy and Environment Change Ideas
DyckFehderau et al (37)	Asset mapping; high bias risk (score, 0)	2 high school students; 7 students in grade 6	Suggested improvements in park and recreation facilities.
Ndirangu et al (46)	Needs assessment; high bias risk (score, 0)	21 community members; 9 university researchers	Suggestions on nutrition and PA in school curriculum; fines or policy for loose dogs; improvement in parks and recreation facilities, walking trails, and street lighting; marketing through television advertisement depicting local community members exercising.
Jilcott Pitts et al (48)	Mixed methods ranking of COCOMO strategies; medium bias risk (score, 2)	336 community members	Most winnable: increasing opportunities for extracurricular PA. Winnable: enhancing infrastructure supporting bicycling and walking. Least winnable: zoning for mixed-use zoning. Government regulations or mandates were not favorably perceived. Rural landscape was a barrier to walkability and locating schools near neighborhoods. Community support for policy change was high for all 7 COCOMO strategies, highest for “communities should improve sidewalks to support walking” and “communities should improve access to outdoor exercise and recreation places.”

Abbreviations: COCOMO, Common Community Measures for Obesity Prevention; PA, physical activity.

PA changes were reported in 14 studies with mixed results; 10 studies reported successfully implementing policy or environmental changes and positive changes in PA. However, PA measurement methods varied across studies, and only 4 studies reported significant positive changes in PA (40,49,51,52). COCOMO strategies were applied in 9 studies reporting increases in PA; 2 studies used pedometers (51,52), 1 study used a combination of self-report and accelerometry (31–34), and 7 studies used self-report or proxy self-report (28,36,38,40,49,50,56). These 9 studies reporting positive changes in PA used at least one of these 5 COCOMO strategies: no. 12, “require PE in schools” (31-34); no. 13, “increase amount of physical activity in PE programs” (31-34); no. 14, “increase opportunities for extracurricular physical activity” (28); no. 15, “reduce screen time in public venues” (36); no. 16, “improve access to outdoor recreation facilities” (51,52,56); and no. 18, “enhance infrastructure for walking” (38,40,49,51,52,56). All but one (56) of these 9 studies also implemented at least one non-COCOMO strategy (28,31–34,36,38,40,49,51,52,): increase amount of and access to PA equipment or improve existing equipment resources (36,38); promote PA resources (38,40,49); adopt worksite policies or practices (28,38); improve access to public buildings after hours for PA purposes (40); reduce sedentary time in school or preschool settings (36); and increase PA opportunities at school outside of PE (31–34,51,52). One of the 10 studies reporting positive PA results implemented only a non-COCOMO strategy: provide access to public buildings after hours for PA purposes (50). Four studies found no change in PA; one of these used both self-report and a subset of accelerometer data (54,55), and 3 studies used self-report or proxy self-report (47,53,57). Of these 4 studies, one used COCOMO strategy no. 12, “require PE in schools” (54,55), 2 studies used strategy no. 13, “increase amount of physical activity in PE programs” (53,57), 3 studies used strategy no. 14, “increase opportunities for extracurricular physical activity”(47,53–55), and one used strategy no. 15, “reduce screen time in public service venues” (53). All but one (57) of these studies also implemented at least one non-COCOMO strategy; one study increased amount of and access to PA equipment or improved existing equipment resources (53), 3 studies increased PA opportunities at school outside of PE (47,53–55), and one reduced home screen time (53).

## Discussion

We found 26 unique studies that implemented PA-related COCOMO or non-COCOMO strategies in rural communities. Given the variation in settings, methods, and results of the studies reviewed, we were unable to empirically assess effectiveness; however, these findings provide a synthesis of current practices and guidance on implementing policy and environmental strategies in rural communities.

Seven of the 12 PA-related COCOMO strategies (nos. 12–18) were applied in 2 or more nonformative studies, suggesting that these strategies are relevant in rural communities. All but 2 studies (29,47) that used these 7 strategies reported effectively implementing them in the target rural communities. Ten studies reported improvements in PA after implementation of policy or environmental changes. “Enhancing infrastructure supporting walking” (no. 18) was implemented in 6 of these 8 studies, with significant changes in 4 studies. However, because 5 of these 6 studies implemented more than one strategy, we cannot attribute the improvement in PA to this strategy alone. Three of the COCOMO strategies were not implemented in any of the reviewed studies, and 2 strategies were implemented in only one study each, suggesting these strategies might be less relevant for rural communities, as originally cautioned when the guidelines were released (21). These strategies relate to location of schools, improvement of public transportation, mixed-use zoning, enhanced personal safety, and traffic safety in areas where people could be physically active. Rural communities may not use these strategies because they lack the resources to implement these strategies or because of other constraints related to small and dispersed populations in comparison with urban and suburban communities. For example, many rural areas have limited or no public transportation systems (59,60) and may not have the tax base or concentration of users to make a public transportation system feasible (61). The studies reviewed implemented 8 non-COCOMO strategies. Although these strategies may not be germane to rural areas only, they have been implemented in rural areas; more research on their effectiveness in rural areas is warranted. Given the increase in policy and environmental approaches for improving PA after the publication of the COCOMO strategies in 2009, our review is beneficial to the field and indicates it may be an opportune time to update PA-related COCOMO strategies (21).

Several relevant studies were published after this review was conducted. A search of literature published from June 2013 through October 2015 found 6 additional articles representing 5 interventions that implemented PA-related policy or environmental strategies in rural areas. Four studies focused on school or preschool children or facilities (62–66); one focused on both the community-at-large and schools (67); none were RCTs; PA levels increased in 3 studies. PA was measured by using pedometers (65,66), direct observation (63), or through a coalition member’s report (67). Four interventions implemented COCOMO strategies: 2 interventions used no. 12, “require PE in schools” (64–66); 2 interventions used no. 13, “increase physical activity in PE” (64–66); 4 interventions used no. 14, “extracurricular physical activity” (62,64–67); one used no. 16, “improve access to outdoor recreation facilities” (67); and one used no. 18, “enhance infrastructure supporting walking” (67). All 5 interventions implemented at least one non-COCOMO strategy: 3 interventions increased amount of PA equipment (62,63,67); 3 interventions increased PA opportunities at school outside of PE (62,64–66); and 3 interventions adopted a district-wide PA-supportive curriculum (62,64–66).

Most policy or environmental strategies implemented in the studies reviewed focused on schools, whether the target population was students, school employees, or community members using the school facilities outside of school time. Use of schools as the focal point for obesity-related interventions aligns with Institute of Medicine recommendations (68). In rural areas where community resources and safe places to be active are limited (59,60,69–71), school facilities and resources (eg, gyms, fields, playgrounds) are often some of the few, if not the only community assets for PA (72). However, many rural areas are consolidating their school districts and building new schools on the outskirts of rural communities or on state highways rather than renovating existing schools or building new schools within municipal domains or current residential areas; this trend may create school grounds that are less accessible for the more populous areas of a rural county (60,71,73,74). Thus, when school-consolidation decisions are made, accessibility of school facilities for PA should be considered.

Recommended COCOMO measurements were not used in the studies reviewed, suggesting that COCOMO measurement approaches might need to be adapted for rural areas. For example, for strategy no. 18, “enhance infrastructure supporting walking,” the suggested COCOMO measurement is miles of paved sidewalks relative to total street miles. In the studies reviewed, enhancing infrastructure for walking included building walking trails and paving sidewalks; thus, miles of paved sidewalks would not capture all possible supportive infrastructure changes. The scale of some suggested COCOMO measurements are too large to be pertinent to smaller communities. For example, the suggested COCOMO measure for no. 13, “increase the amount of physical activity in PE programs in schools,” is whether the largest school district in the local jurisdiction has a policy that requires K through 12 students to be physically active for at least 50% of PE time. Small school districts, common in rural areas, may not be able to provide the level of detail necessary to determine success using this metric, and rural communities may have only one or 2 school districts; an appropriate rural metric could be the percentage of schools in a district that require K through 12 students to be physically active for at least 50% of PE time. Creating standard valid, reliable, specific, appropriate, and feasible metrics for policy and environmental strategy measurement for rural communities would help these communities better assess the success of policy and environmental strategies and help build an evidence base in rural communities.

Measurement of PA outcomes in the studies reviewed was rare and lacked consistency and methodological strength, limiting interpretation. When PA change was reported, most studies used a form of self-report. Few studies used objective measurement, and those that did measure PA objectively only did so in a subset of their sample, with half using pedometers. Accelerometers are a valid, reliable, and practical measure of PA and are used nationally and internationally (75). Rural evaluations need to consistently measure PA across studies using accelerometers to allow for better understanding of intervention effectiveness and comparison across the urban–rural continuum. Because of the decreasing costs of accelerometers and the ability to borrow units or purchase used units, recent rural community-based approaches have used accelerometers to measure PA and suggested strategies to improve feasibility, accuracy, and consistency (eg, text/email “wear” reminders, data collection methods, scoring methods) for using them in rural communities (75–77).

Lack of detail on study methods and variation in study design, measurement of outcomes, and context limited our ability to compare results of strategies across studies and examine effectiveness. Most studies were biased across assessment categories, indicating overall weakness in research design. Only 14 studies measured change in PA in response to policy or environmental strategies, and measurement approaches greatly varied. Future studies should incorporate elements of strong study design, such as clearly defined inclusion or exclusion criteria, protocols for data collection and use of measurement tools, measurement of potential confounders, reporting of sample size and estimated reach, and objective measurement of change in PA behavior.

Despite the challenges discussed and the challenges inherent in the subjective methods of systematic reviews, this review and its companion (24), provide a synthesis of the data on the use of COCOMO strategies in rural communities. The main findings of both reviews include the importance of making schools the focal point of nutrition- and PA-related interventions and building on existing community resources. Additionally, several nutrition- and PA-related COCOMO strategies, such as improvement of public transportation or geographic availability of supermarkets, may not be applicable to rural communities. We recommend inclusion of non-COCOMO PA-related strategies and refinement of current COCOMO recommended measurements. Improvements for current COCOMO nutrition-related strategies have been suggested (24). Both reviews recommend the use of stronger study design and measurement of policy, environment, and behavior in future studies (24). We echo a conclusion that additional guidance on implementation of policy or environmental strategies in rural communities could be found in research not published in scientific literature (eg, websites, gray literature) (24). Although we used many strategies to identify studies conducted in rural settings, the definition of “rural” varies (71), and studies that were not explicitly identified as rural by their authors might not have been included. Although the variability in rural communities (by geography, population density, topography, resources, and other factors) should be considered in obesity prevention approaches (71), these reviews described strategies that were successfully implemented in multiple rural communities.

COCOMO strategies and recommended measurements provide an evidence-based approach to address obesity and measure the success of intervention strategies. Most PA-related strategies appeared to be applicable in rural communities; however, measurements recommended by COCOMO were not always appropriate. Several non-COCOMO strategies were effectively implemented in rural communities. Generating a database of recommended strategies and measurements relevant to rural communities should be considered. Further research, using robust study designs and measures, is needed to better ascertain the success and effectiveness of implementing policy and environmental strategies in rural communities. This information could aid policy makers and community leaders in decision making on resource allocation and obesity-prevention efforts in their rural communities.
